# Current Trends in Proteomic Advances for Food Allergen Analysis

**DOI:** 10.3390/biology9090247

**Published:** 2020-08-25

**Authors:** María López-Pedrouso, José M. Lorenzo, Mohammed Gagaoua, Daniel Franco

**Affiliations:** 1 Department of Zoology, Genetics and Physical Anthropology, University of Santiago de Compostela, 15872 Santiago de Compostela, Spain; mariadolores.lopez@usc.es; 2 Centro Tecnológico de la Carne de Galicia, Rúa Galicia N° 4, Parque Tecnológico de Galicia, 32900 San Cibrao das Viñas, Spain; jmlorenzo@ceteca.net; 3 Área de Tecnología de los Alimentos, Facultad de Ciencias de Ourense, Universidad de Vigo, 32004 Ourense, Spain; 4 Food Quality and Sensory Science Department, Teagasc Ashtown Food Research Centre, Dublin 15 Ashtown, Ireland; gmber2001@yahoo.fr

**Keywords:** allergomics, proteins, immunoglobulin E, selected reaction monitoring (SRM), multiple reaction monitoring (MRM), immunoblotting, cross-reactivity, gluten, parvalbumin, myosin and tropomyosin

## Abstract

Food allergies are a global food challenge. For correct food labelling, the detection and quantification of allergens are necessary. However, novel product formulations and industrial processes produce new scenarios, which require much more technological developments. For this purpose, OMICS technologies, especially proteomics, seemed to be relevant in this context. This review summarises the current knowledge and studies that used proteomics to study food allergens. In the case of the allergenic proteins, a wide variety of isoforms, post-translational modifications and other structural changes during food processing can increase or decrease the allergenicity. Most of the plant-based food allergens are proteins with biological functions involved in storage, structure, and plant defence. The allergenicity of these proteins could be increased by the presence of heavy metals, air pollution, and pesticides. Targeted proteomics like selected/multiple reaction monitoring (SRM/MRM) have been very useful, especially in the case of gluten from wheat, rye and barley, and allergens from lentil, soy, and fruit. Conventional 1D and 2-DE immunoblotting have been further widely used. For animal-based food allergens, the widely used technologies are 1D and 2-DE immunoblotting followed by MALDI-TOF/TOF, and more recently LC-MS/MS, which is becoming useful to assess egg, fish, or milk allergens. The detection and quantification of allergenic proteins using mass spectrometry-based proteomics are promising and would contribute to greater accuracy, therefore improving consumer information.

## 1. Introduction

Food allergies represent a major problem in our society. The main allergens consist of a group of proteins that trigger an abnormal response by immunoglobulin E (IgE). In general, cellular mechanisms that are mediated, non-mediated by IgE, or a combination of both, involving gastrointestinal tracts can induce allergic reactions [1]. Approximately 5% of adults and 8% of children are affected by food allergies in western countries, entailing a high economic cost for the health system [2]. In fact, food allergies have been estimated to cost $24.8 billion annually in the USA, including direct medical costs and charges borne by the family [3]. Recent data on the prevalence of the population indicate that food allergies are growing, and this is mainly associated with industrialization in developing countries [4]. On the contrary, a recent study reported that evidence of a rising prevalence in developing countries could be associated with their economic expansion [5]. From 1997 to 2007, the prevalence of reported food allergies increased by 18% among children under 18 years [6], meanwhile, in adults, the prevalence increased significantly in 2010 from 9.1% to 13% compared to 2001 [7]. Moreover, food intolerances caused by non-immune mechanisms are much more common, with a prevalence of 3 to 5 times higher than allergies. For example, Nwaru et al. [8] indicated that food hypersensitivity in adults ranges from 3.5 to 20%, with the greatest and lowest prevalence occurring in Northern and Eastern Europe, respectively. Both hypersensitivities and allergies can lead to different symptoms, resulting in the difficulty of their differentiation [9]. Further, the prevalence of these health problems is expected to increase in the coming years with industrialization [10]. Although several treatments have been tested or proposed, it is known that the most effective method is not to consume allergenic foods. However, this is very difficult to follow due to the wide distribution of allergens and defects of labelling of many processed products containing several ingredients (e.g., gluten, lactose, proteins and other components of milk, egg, dried fruit, soy, etc.) which are also a source of allergies. Hence, the detection of food allergens is highlighted as a challenge, encouraging technological efforts [11].

Based on the above, the food industry is constantly developing to meet the market needs and the quest for healthy and ready-to-eat products that would prevent food allergies and intolerances. For all these reasons, allergen detection and characterization in fresh and processed foods are the main objectives of the food processing industry sector. In most cases, the allergies are produced by “big 8” major food allergens: peanut, milk, egg, soy, wheat, fish, shellfish, and tree nuts. These ingredients should be properly labelled in accordance with the Food and Drug Administration (FDA) [12], and even in the EU allergen labelling requirements include 14 allergens [13]. Apart from these ingredients, cross-contamination is an additional risk due to an allergic reaction occurring in the presence of a trace amount of the substance. Novel sources of food, as well as new ways of processing, require a thorough search for new allergens [14]. Therefore, the most accurate, sensitive, and fast analytical tools are increasingly demanded for allergen food control. The potential of OMICS technologies in this field is enormous, allowing high accuracy of detection and quantification of food allergens [15,16,17].

**Figure 1:**
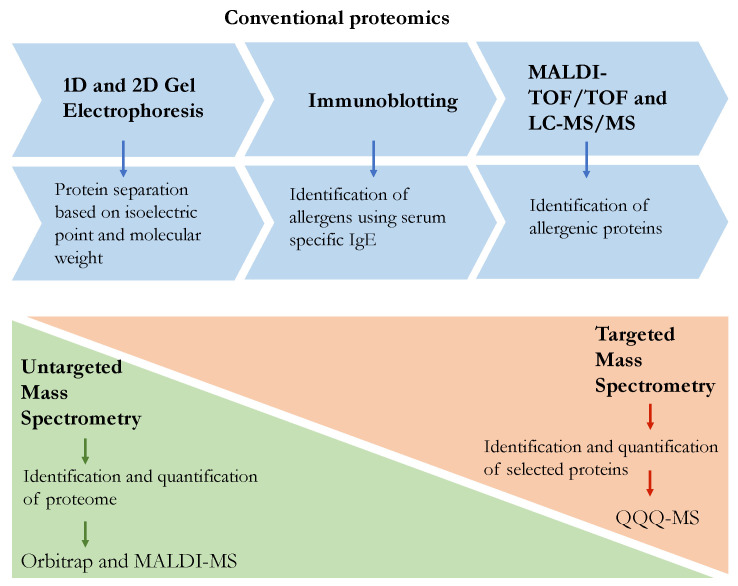
Key elements of proteomic approaches employed in allergenicity assessment. The blue color indicates conventional proteomics, and orange and green highlight targeted and untargeted mass spectrometry, respectively. Figure acronyms: 1D and 2D gel electrophoresis = one and two dimensional gel electrophoresis; TOF = time of flight; MALDI-TOF/TOF = Matrix-assisted laser desorption ionisation-tandem mass spectrometry (TOF); LC-MS/MS = liquid chromatography-tandem mass spectrometry; QQQ-MS = triple quadrupole mass spectrometer.

Proteomics, and more specifically proteomics-based tools using mass spectrometry, are increasingly used in the field of food quality and safety [18,19,20]. In Figure 1, the most relevant aspects of proteomic methods and techniques are summarised. For instance, new applications such as authentication, allergen detection, or identification of gluten fractions associated with celiac disease are being developed [21]. The detection and quantification of food allergens remain major challenges because the antibody-based assays are limited by matrix/processing effects and epitope masking. These constraints are overcome by mass spectrometry [22]. A proteomic approach can be used to characterize new food allergens in the food matrix. For example, the conventional 2-DE analysis followed by Western immunoblotting with sera from allergic patients and mass spectrometry analysis leads to the identification of new allergens [23]. Traditionally, immunoassays, such as the enzyme-linked immunosorbent assay (ELISA), which rely on the recognition of monoclonal or polyclonal antibodies have been widely used. However, ELISA has several disadvantages due to food protein denaturation and epitopes degradation during the thermal process, causing a weak sensitivity of ELISA assays. To this end, technological advances provided several ELISA kits based on antibodies targeted against denatured proteins. As a consequence, immunoassays such as ELISA and Western blotting are now replaced by the targeted MS/MS techniques and data independent acquisition (DIA) methods allowing better selectivity, precision, and accuracy of quantification [24]. Within MS techniques, the targeted proteomics represents another significant approach to monitor a specific set of proteins using selected or multiple reaction monitoring (SRM/MRM) by quadrupole-orbitrap (Q-orbitrap). In the case of unknown proteotypic peptides, shotgun proteomics can be carried out, resulting in a fingerprint for targeted proteomics investigations [25]. Available information of proteotypic peptides for each allergen provides a great resource and a suitable scenario for the identification at any food matrix and sample preparation [26]. A key aspect to be able to develop this approach is that more intensive efforts are needed to investigate due to the complexity of the food/matrix, the modifications of the peptides, and the search for specific peptides for a given allergen [27]. Furthermore, the safety of novel foods and the characterization of potential protein-based allergens is needed, and mass spectrometry methods could be applied to harmonize and validate the allergen data [28].

The aim of this review is to provide the latest advances from published literature during the last five years related to proteomic techniques applied for allergen determination and characterization. A collection of current proteomic studies about the most common allergens in vegetable and animal food products is given. This review focuses on the current technological advances and their advantages as well as a brief discussion on the drawbacks of the applied methods and techniques.

## 2. Food Allergens in Vegetable and Animal Products

The potential of advanced proteomic techniques like reliable and sensitive methods for detecting and identifying the allergens depends greatly on the type of the matrix [29]. It is worth mentioning that allergens may be embedded into food ingredients at trace levels, provoking possible discrepancies between food content and labelling. Additionally, a wide variety of isoforms and post-translational modifications (PTMs), as well as structural changes during processing, could determine the allergenicity of the proteins. The heterogeneity of allergens is reflected in AllerBase (http://bioinfo.unipune.ac.in/AllerBase/Home.html), which is a structural database of allergens with 2137 experimentally validated allergens and 3D structures from animals, plants, fungi, bacteria and viruses [30]. Hence, a new specific area arises, allergomics, which focuses on food allergen and high-throughput technologies for their systematic analysis, such as targeted proteomic analysis for the detection of IgE-binding proteins [31,32].

### 2.1. Plant-Based Food Allergens

Plant-based proteins are of great importance as protein sources in non-developed countries due to their lower cost and the growing number of vegetarians in developed countries. However, the main concern is that many allergies from plant origin interact with the human body. The main allergic vegetal foods regulated by the EU are peanuts, soy, tree nuts, and wheat. However, there are other sources of allergens, such as legumes, fruits, and vegetables (see Table 1). There is a new trend of producing healthy vegetable food to meet vegetarian and vegan demand. Nowadays, ethical issues about animal production, animal welfare aspects, and environmental concerns of meat production and consumption in developing countries are considered by consumers. New emerging technologies such as texturization processing by extrusion have come up to suggest new vegetal products imitating meat products. However, the main problem of this strategy is the allergenicity caused by vegetable foods. Most of these allergens are proteins with different biological functions involved in storage, structure, and plant defence, which may be altered by biotic or abiotic environmental stresses. It has been proved that chemical pollution caused by heavy metals, air pollution and pesticides triggered the expression of allergens [33]. On the other hand, modern biotechnology increasingly develops genetically modified (GM) crops in order to increase the production or other agronomic aspects, hence causing modifications in the allergenicity of the new food ingredients. In this context, the *Codex Alimentarius* Commission encouraged including an investigation of allergenic tendencies of GM crops [34]. In the case of soy, the European Food Safety Authority (EFSA) has found no evidence that GM soy differed in allergen content [35]. This implies a difficult challenge from a technical point of view to identify and quantify vegetal allergens.

Particularly in plant organisms, targeted proteomics including selected/multiple reaction monitoring (SRM/MRM) and data-independent acquisition DIA method or sequential window acquisition of all theoretical fragment ion spectra mass spectrometry (SWATH-MS) offer an alternative to traditional biochemical methods [36]. These emerging approaches allow both the identification and quantification of allergens in a broad range of plants (see Table 1). These new tools mainly focus on the detection and quantification of proteotypic peptides used as markers of the specific allergen, therefore overcoming limitations such as complex matrices, extraction difficulties, and incomplete sequence information [37]. Another classic proteomic approach, 2-DE followed by Western immunoblotting and MS, remains the gold standard method. It is worth mentioning that the main limitations of 2-DE are the low reproducibility, difficulties in the study of hydrophobic proteins, and basic proteins and low dynamic range, but these constraints are overcome by the use of new technological developments such as immobilized pH gradient (IPG) strips [23].

Celiac disease, gluten hypersensitivity, wheat allergy, and wheat-dependent exercise-induced anaphylaxis (WDEIA) could be induced by proteins from wheat, barley, and rye. The most abundant gluten proteins are glutenins and gliadins, which play an important role in viscoelasticity of dough and final products. These proteins, defined as prolamins with high contents of proline and glutamine, are responsible for celiac disease and non-IgE mediated food intolerance. Several proteomic analyses have been developed aiming to identify and quantify the gluten proteins from processing products ensuring gluten-free foods. Hence, special efforts were made to provide a curated gluten protein sequence database to support proteomic technologies [38]. As in the case of other crops, wheat quality was constantly enhanced due to genetic improvement programmes causing modifications at the genotype level. The knowledge of the major gluten protein genes of cultivars will also enhance the detection and identification of these allergens from a proteomic point of view [39]. Even the preparation of reference materials of gluten proteins from wheat, rye, and barley flours were further considered and their characterization using MRM was carried out [40].

Other important groups of food allergy are produced by legumes, nuts, and seeds, causing an anaphylactic reaction in some cases. The allergenic proteins are mainly proteins of storage, such as 2S albumins, 7S globulins, and 11S globulins with high resistance to heat and gastrointestinal enzymes. The lentic cultivars as other species are modified to improve the quality traits and decrease allergen proteins. An earlier proteomic study used MRM to characterize the allergens in lentil cultivars by means of peptides from vincilin, legumin, lectin and lipid transfer proteins [41]. Similarly, the variations in allergen content from cultivars of soy were considered by SRM based on storage proteins [42]. Therefore, it seems that shotgun proteomics could be a suitable choice to select the cultivar with lesser allergen proteins in lentil seed. The allergenicity of soy protein isolate could be reduced by high hydrostatic pressure. Indeed, significant changes were produced in 7S and 11S globulin, hence reducing the allergenicity in children [43]. However, Ribeiro et al. [44] found minor differences of allergenic potential of thirteen hazelnut (*Corylus avellana* L.) varieties. The most abundant IgE-reactive proteins, Cor a 9 and Cor a 1.04, were identified by 2-DE.

Other allergenic foods that have been widely studied are fruits and vegetables. Most adverse reactions to fruits and vegetables are associated with pollen allergies because of “cross-allergenicity”. The cross-reaction of human antibodies occurs against pollen allergens at the molecular level. In the case of birch pollen allergic patients, we count between 50 and 93% of patients [45]. It important to note that allergen activity of most fruits and vegetables is reduced with heating and processing. To determine the identification and quantification of the allergen trace, a signature peptide can be used to study the stability of the allergen in different fruit foods. For instance, in the case of kiwi allergens, kiwi jam, hot-air-dried kiwi, lyophilized kiwi, pasteurized puree, high-pressure-processed puree, pasteurized juice, and juice were analysed, resulting in the content of Act d 1 and Act d 5 lower in jam and hot-air-dried kiwi than in the other kiwi products (Table 1). As small amounts of these allergens may cause a strong reaction of the human body, the low abundance of allergens should be investigated using enrichment methods. In mango and banana proteomes, new allergens, as well as others which are better known, could be detected using the combinatorial peptide ligand library [46,47].

The peanut allergy is a major concern in developed countries, causing severe health problems. The patient has only one solution: avoid its consumption. In this context, unfortunately, there is a large list of foods containing peanuts or peanut oil. The main allergens of peanut are seed storage proteins (7S and 11S globulin), prolamin family members (2S albumins), and lipid transfer proteins. Using LC-MS, these allergens could be detected in food ingredients for oral meals, which might be used for setting a criterion of acceptance or rejection. The most abundant allergens, Ara h 1 and Ara h 3, were quantified by MS-based analysis employing DIA coupled with ion mobility MS [48]. Moreover, the stability of 2S albumins plays a key role in the disease process as well as in the in vitro digestion studies supported by 2-DE immunoblotting and nLC-MS/MS. Thus, the major peanut allergens remain unaltered after oral and gastric digestion, causing serious allergic reactions [49].

### 2.2. Animal-Based Food Allergens

Food allergens from animal food products are mainly from milk, eggs, and fish, as reflected in current regulations. Allergies caused by meat are quite rare, but several meat allergies have been described in young atopic children. Most cases are related to specific IgE to galactose-α-1,3-galactose, an oligosaccharide of non-primate mammals [52]. From a proteomic point of view, red meat allergy against the carbohydrate α-Gal epitope has been investigated [53]. Consequently, we will focus on other food allergies related to animal-based protein allergens.

Egg allergy is a serious health problem that mainly affects children. Eggs consist of two differentiated soluble parts, the egg white and the egg yolk. It has been evidenced that egg white is the major source of allergenicity, and the most common proteins are ovomucoid (Gal d1), ovalbumin (Gal d2), ovotransferrin (Gal d3), and lysozyme (Gal d4). In the yolk, two major allergens were detected: α-livetin (Gal d 5) and YGP42 (Gal d 6) [54]. From a proteomic point of view, the stability and the enzymatic digestion on the allergenicity of egg white were investigated, resulting in the lysozyme, a highly immunoreactive protein unaltered by enzymatic digestion.

Seafood includes edible aquatic animals such as fish, crustaceans, and molluscs. These organisms produce many allergenic proteins resulting in the difficulty to identify and characterize all of them accurately. For seafood, the allergens with a major allergenic response are parvalbumin, tropomyosin, and arginine kinase, inducing immunological and clinical cross-reactivity [55]. Most of the allergic reactions are produced by parvalbumins and, for this reason, parvalbumins are the most investigated by proteomics studies (see Table 2). On another hand, farmed fish are mostly fed with different diets to reduce the allergenicity, such as EDTA-enriched diets allowing the removal of calcium ions from β-parvalbumins. This approach reduces the interaction between β-parvalbumins and IgE [56]. Moreover, the parvalbumin content in fish muscle could be reduced by feeding strategies, for example, with different creatine percentages [57]. In both cases, the content of parvalbumin and the identification of their isoforms can be easily monitored by either 1D or 2-DE immunoblotting using patient sera. Aiming to identify a potential peptide vaccine, shotgun proteomics was used for the characterization B-cell epitopes of β-parvalbumins. This strategy could be used for the design of new potential immunotherapies [58]. Nowadays, other novel allergens and the immunological cross-reactivity of the known allergens in shellfish are researched using both transcriptomics and proteomics [59].

Milk allergy is a common abnormal response of the immune system of infants to cow milk and products containing milk. The cow milk allergy is the most frequent, but milk from sheep, goats, and other mammals may cause allergies. The main allergenic proteins in milk are α-lactalbumin (also called Bos d 4), β-lactoglobulin (Bos d 5), and casein (Bos d 8) based on IgE measurements. However, other minor proteins, such as lactoferrin, bovine serum albumin (BSA), and immunoglobulins could also be immune-reactive proteins [60]. In such cases, the products containing milk should be labelled and avoided by allergenic patients. Rapid immobilized trypsin digestion combined with UPLC-MS/MS has been proven to be very effective for the detection of αs1-casein, αs2-casein, β-casein, and κ-casein in baked food [61]. Additionally, milk is consumed after a thermal treatment with the purpose of prolonging shelf-life and preserving microbiological safety. The pasteurization and ultrahigh-temperature treatments may affect the digestibility of modified proteins. This thought to happen through a Maillard reaction due to the resistance of the proteins to digestion, hence producing a major allergenic response. Moreover, glycation and glycoxidation of Maillard products can modulate the allergenic impact [62,63]. A proteomic approach, as well as an immunoblot analysis of pasteurized and baked milk, led to an allergenic response with respect to unprocessed milk. From these studies, it has been demonstrated that heating treatment together with other food components could reduce the allergenicity of cow milk [64]. In the case of whey proteins, the loss of the allergenic effect is produced by heating it for 30 min at 65 °C or above [65]. Additional studies were focused on the search of peptides for the detection of casein and whey milk allergens from baked cookies containing known amounts of non-fat dry milk. In fact, nineteen candidate peptides of four casein proteins and two whey proteins were identified using PRM [66].

Nowadays, insects represent an alternative source of proteins to overcome the increasing world population. It has been demonstrated that silkworm, mealworm, caterpillars, *Bruchus lentis*, sago worm, locust, grasshopper, cicada, bee, *Clanis bilineata*, and the food additive carmine are sources of allergies. The allergens tropomyosin and arginine kinase were identified in insects as well as in crustaceans and house dust mites [67]. Using shotgun proteomics, peptides could be identified from the lesser mealworm and black soldier fly. Enzymatic hydrolysis was used as an appropriate treatment to reduce the allergenic risk in lesser mealworms [68]. On the other hand, arginine kinase from mealworms and crickets commercially available was assessed, showing a low specific reactivity of this allergen [69].

## 3. Conclusions

Great effort and innovation in proteomics and based-methods have been carried out for the study and control of food allergies. Thanks to these high-throughput methods, the search and identification of new isoforms of allergens have been determined in different food matrixes. However, standardized methodologies and robust methods of assessments will be necessary in the near future for accurate and fast detection at the food industry level. It is necessary to routinely detect and quantify allergenic proteins, and in this regard, mass spectrometry-based proteomics is a powerful tool. Very little information is available about the post-translational modifications of allergenic proteins, but they play a fundamental role in protein allergenicity, and exciting research is expected in the future of this field. Curated allergen databases to classify proteins to identify risks, the IgE-binding epitope, or the organism source are required. These technological aspects will be solved in the coming years and food products will become healthier for different groups of targeted consumers.

## Figures and Tables

**Table 1:** A non-exhaustive list of current proteomics technologies for food allergen detection and quantification in plant-based foods.

Food	Allergen	Allergenic Protein	Proteomic Technology	Aim	Ref.
Cereals containing gluten					
Wheat Rye Barley	-	Gliadins (wheat)	MRM by LC-MS/MS	I, Q	[40]
		Secalins (Rye)			
		Hordeins (Barley)			
Wheat	-	Omega-5 gliadin	2-DE immunoblotting	I	[39]
		Gamma gliadins			
**Legumes and soy**					
Lentil	Len c 1 Len c 2	Vincillin Legumin Lectin Lipid transfer proteins	MRM by LC–MS/MS	I, Q	[41]
Soy	Glycinin G1 Glycinin G2 Glycinin G3 Glycinin G4	Glycinin	SMR by LC-MS/MS	I, Q	[42]
	Gly m Bd 28k	Vincilin			
	Gly m Bd 30k	Cysteine protease			
	Β-Conglycinin α subunit	Vincilin			
	Kunitz Trypsin Inhibitor 1 Kunitz Trypsin Inhibitor 3	-			
**Fruits**					
Mango	Man i 1	Glyceraldehyde 3-phosphate dehydrogenase	2-DE immunoblotting followed by nano LC-MS/MS	I	[46]
	Man i 2				
	Mus a 1	Profilin			
	Mus a 2	Class 1 chitinase			
	Mus a 5	Beta-1-3-glucanase			
	-	Pectinesterase			
	-	Superoxide dismutase			
Kiwifruit	Act d 1	Actinidin	MRM by LC-MS	I, Q	[50]
	Act d 5	Kiwellin			
	Act d 11	Ripening-related protein family			
Goji Berry Superfruit	-	Vincilin	1D immunoblotting followed by LC-MS/MS	I	[51]
		Legumin			
		11S globulin			
Banana	Mus a 7	Catalase	2-DE immunoblotting and LC-MS/MS	I	[47]
**Peanuts**					
Peanut	Ara h 1 Ara h 3	Cupin	LC-MS	I, Q	[48]
	Ara h 5	Profilin			
	Ara h 2 Ara h 6 Ara h 7 Ara h 9	Prolamin			
	Ara h 10 Ara h 11	Oleosin			
Peanut	Ara h 1 Ara h 3	Cupin	1D and 2-DE immunoblotting and nLC-MS/MS		[49]
	Ara h 2 Ara h 6.	Prolamin			

I = identification, Q = quantification.

**Table 2:** A non-exhaustive list of current proteomics technologies for food allergen detection and quantification in animal foods.

Food	Allergen	Allergenic Protein	Proteomic Technology	Aim	Ref.
Eggs					
Hen egg	Gal d 1	Ovomucoid	1D and 2-DE immunoblotting and MALDI-TOF/TOF	I	[70]
	Gal d 2	Ovalbumin			
	Gal d 3	Ovotransferrin			
	Gal d 4	Lysozyme			
**Fish and shellfish**					
Ray’s bream White seabream Cod Pink cusk-eel Four-spot megrim Angler Deep-cape hake Common seabream Salmon Club mackerel Common sole Gilthead seabream Yellowfin tuna Horse mackerel Swordfis	-	β-parvalbumin	Shotgun proteomics	I	[58]
Gilthead seabream	-	β-parvalbumin	1D and 2-DE immunoblotting and MALDI-TOF/TOF	I	[56]
Cod Flounder Hake Herring Mackerel Bass Tuna Trout Salmon	-	Parvalbumin Tropomyosin Aldolase α β-enolase Collagen	1D and 2-DE immunoblotting and LC-MS/MS	I	[71]
Gilthead Seabream	-	Parvalbumin	2-DE immunoblotting and MALDI-TOF/TOF	I	[57]
Barramundi Salmon, Tuna	-	Collagen α chains	LC-MS/MS	I	[72]
Whiteleg shrimp	-	Arginine kinase Myosin light chain Pyruvate kinase Tropomyosin	1D and RP-nano-UPLC- ESI-MS/MS	I	[73]
Shrimp allergy	-	Tropomyosin	1D immunoblotting and LC-MS/MS		[74]
**Milk**					
Cow’s milk and muffins	Bos d 5	β-lactoglobulin	SDS-PAGEUPLC coupled with HR-MS/MS	I	[64]
	Bos d 4	α-lactalbumin			
	Bos d 6	Serum albumin			
	Bos d 7	Inmunoglobulins			
	Bos d 9	αS1-casein			
	Bos d 10	αS2-casein			
	Bos d 11	β-casein			
	Bos d 12	κ-casein			
Baked food	Bos d 9	αs1-casein	UPLC-MS/MS	I	[61]
	Bos d 10	αs2-casein			
	Bos d 11	β-casein			
	Bos d 12	κ-casein			
Cow’s milk	-	Whey proteins	1D and LC-MS/MS	I	[65]
**Alternative foods**					
Lesser mealworm Black soldier fly	-	Actin Tropomyosin Myosin	1D immunoblotting and LQT-orbitrap	I	[68]
Mealworms Crickets	-	Arginine kinase	MALDI	I	[69]
Macroalgae Ulva sp.	-	Superoxide dismutase Troponin C Aldolase A Thioredoxin h	LC-MS/MS	I	[75]

I = identification.
